# Spatial Heterogeneity of Cx43 is an Arrhythmogenic Substrate of Polymorphic Ventricular Tachycardias during Compensated Cardiac Hypertrophy in Rats

**DOI:** 10.3389/fcvm.2016.00005

**Published:** 2016-03-02

**Authors:** Mohamed Boulaksil, Marti F. A. Bierhuizen, Markus A. Engelen, Mèra Stein, Bart J. M. Kok, Shirley C. M. van Amersfoorth, Marc A. Vos, Harold V. M. van Rijen, Jacques M. T. de Bakker, Toon A. B. van Veen

**Affiliations:** ^1^Interuniversity Cardiology Institute of the Netherlands, Utrecht, Netherlands; ^2^Department of Medical Physiology, Division of Heart and Lungs, University Medical Center Utrecht, Utrecht, Netherlands; ^3^Department of Cardiology, Radboud University Medical Center, Nijmegen, Netherlands; ^4^Division of Cardiology, Department of Cardiovascular Medicine, University of Muenster, Muenster, Germany; ^5^Division of Heart and Lungs, Department of Cardiology, University Medical Center Utrecht, Utrecht, Netherlands; ^6^Heart Failure Research Center, Academic Medical Center, Amsterdam, Netherlands

**Keywords:** arrhythmia, sudden death, electrophysiology, hypertrophy

## Abstract

**Background:**

Ventricular remodeling increases the propensity of ventricular tachyarrhythmias and sudden death in patients. We studied the mechanism underlying these fatal arrhythmias, electrical and structural cardiac remodeling, as well as arrhythmogeneity during early, compensated hypertrophy in a rat model of chronic pressure overload.

**Methods:**

Twenty-six Wistar rats were subjected to transverse aortic constriction (TAC) (*n* = 13) or sham operation (*n* = 13). Four weeks postoperative, echo- and electrocardiography was performed. Epicardial (208 or 455 sites) and transmural (30 sites) ventricular activation mapping was performed on Langendorff perfused hearts. Subsequently, hearts were processed for (immuno)histological and molecular analyses.

**Results:**

TAC rats showed significant hypertrophy with preserved left ventricular (LV) function. Epicardial conduction velocity (CV) was similar, but more dispersed in TAC. Transmural CV was slowed in TAC (37.6 ± 2.9 cm s^−1^) compared to sham (58.5 ± 3.9 cm s^−1^; *P* < 0.01). Sustained polymorphic ventricular tachycardias were induced from LV in 8/13 TAC and in 0/13 sham rats. During VT, electrical activation patterns showed variable sites of earliest epicardial activation and altering sites of functional conduction block. Wandering epicardial reentrant activation was sporadically observed. Collagen deposition was significantly higher in TAC compared to sham, but not different between arrhythmogenic and non-arrhythmogenic TAC animals. Connexin43 (Cx43) expression was heterogeneous with a higher prevalence of non-phosphorylated Cx43 in arrhythmogenic TAC animals.

**Conclusion:**

In TAC rats with compensated cardiac hypertrophy, dispersion of conduction correlated to arrhythmogenesis, an increased heterogeneity of Cx43, and a partial substitution with non-phosphorylated Cx43. These alterations may result in the increased vulnerability to polymorphic VTs.

## Introduction

During cardiac pathophysiology, both the compensated and decompensated heart is characterized by structural and electrical remodeling, which is accompanied by a high vulnerability to ventricular arrhythmias and sudden cardiac death (1–4). Cardiac remodeling may be associated with conduction slowing, which increases the propensity for reentrant arrhythmias (5). Not necessarily related to conduction slowing, in addition, local heterogeneous differences in impulse conduction due to spatial differences in remodeling may lead to increased dispersion of impulse conduction, which is proarrhythmogenic as well (6, 7).

Impulse conduction in ventricular myocardium is primarily determined by 1) sodium current (I_Na_), mainly directed by the Na_v_1.5 sodium ion channel protein, 2) tissue architecture, primarily determined by collagen deposition and cell size, and 3) cell-to-cell coupling, which is mainly mediated in ventricular myocardium by channels that is composed of the gap junction protein connexin43 (Cx43) (5).

Currently, it is unknown to what extent abnormal conduction characteristics contribute to arrhythmogenesis in early, compensated stages of cardiac remodeling. Several studies have shown that abnormal distribution of Cx43 is a key feature of the hypertrophied heart (8, 9). In that respect, heterogeneous distribution of Cx43 resulted in conduction defects and ventricular arrhythmias in a chimeric mouse model (10). In more advanced stages of cardiac disease, heterogeneous distribution of Cx43 has strongly been associated with the occurrence of arrhythmias in both patients and mice (11, 12). Adding to this aspect of heterogeneous remodeling, Cx43 is a phosphoprotein with several phosphorylation states, which determine its gating properties. Both phosphorylating protein kinases and dephosphorylating protein phosphatases are modulators of Cx43 phosphorylation. Dephosphorylation of Cx43 accounts for reduced intercellular coupling that may slow conduction and enhance arrhythmia susceptibility (13–16). It is not clear, however, whether heterogeneous expression of Cx43 is accompanied by, or even results from, a heterogeneous process of Cx43 dephosphorylation. Therefore, we hypothesized that in compensated stages of cardiac remodeling, inhomogeneous dephosphorylation of Cx43 and heterogeneous spatial differences in Cx43 distribution would primarily lead to more dispersed ventricular impulse conduction and an increased propensity for ventricular arrhythmias. In order to acquire in-depth knowledge in this respect, the current study addressed our hypothesis through determination of characteristics of epicardial and transmural impulse propagation and distribution/status of Cx43 gap junction channels.

## Materials and Methods

### Rat Model of Cardiac Hypertrophy

Animal experiments were performed according to institutional guidelines and the Dutch Experiments on Animals Act and approved by the local Animal Experiments Committee. The study complies with the *Guide for the Care and Use of Laboratory Animals* published by the US National Institutes of Health (No. 85-23, revised in 1996). Wistar rats were housed at 21°C and 60% humidity with an artificial 12:12 h light–dark cycle and were fed with *ad libitum* standard chow and water.

Twelve-week-old female Wistar rats were transverse aortic constriction (TAC) (*n* = 13) or sham (*n* = 13) operated, as described previously (11).

### Echocardiography and ECG

To study compensated remodeling, 4 weeks after TAC or sham operation, echocardiography was performed to assess 1) the extent of aortic constriction, 2) left ventricular (LV) dimensions and wall thickness, and 3) LV function. After a mild isoflurane anesthesia, rats were placed in supine position to record an ECG.

### Isolation of the Hearts, Recording of Electrograms, and Arrhythmia Susceptibility Testing

Four weeks after operation, hearts were extracorporated for Langendorff perfusion, and extracellular electrograms were recorded with a 247 or 208 point multiterminal electrode (19 × 13 and 16 × 13 grid, respectively). Recordings of LV and right ventricle (RV) were made during stimulation from the center of the grid on LV at a basic cycle length (BCL) of 150 ms. To map ventricular arrhythmias in both ventricles simultaneously, in 4 of the 13 TAC and 5 of the 13 sham-operated rat hearts, simultaneous epicardial recordings were made from LV (19 × 13 grid) and RV (16 × 13 grid). In the remaining hearts, LV and RV were mapped sequentially with the 19 × 13 electrode.

Conduction velocity (CV) parallel (θ_L_) and perpendicular (θ_T_) to fiber direction was determined from the paced activation maps. Dispersion of epicardial conduction was assessed by calculating the maximal difference at each recording site with neighboring activation times (so-called phase differences). Local phase differences were plotted in a phase map, showing the spatial distribution of inhomogeneity’s in conduction, so-called dispersion in conduction. From each map, a total index of dispersion was calculated (17). Transmural CV (θ_TM_, perpendicular to epicardium) was calculated from local activation times derived from electrograms recorded with three separate needle electrodes.

The susceptibility for arrhythmias was tested by programed stimulation from the center of the grid electrodes. Subsequently, hearts were divided into arrhythmogenic (TAC+) and non-arrhythmogenic (TAC−). Details on recording and analysis of electrograms, and testing of arrhythmia susceptibility were described previously (11).

### (Immuno)Histology and Cx43 Protein Analysis

Following electrical recordings during Langendorff perfusion, hearts were immediately frozen in liquid nitrogen and stored at −80°C. TAC (*n* = 8) and sham (*n* = 6) hearts were sectioned serially in 10 μm slices parallel to the epicardial surface. All slices were mounted on AAS (aminopropyltriethoxysilane)-coated glass slides.

The amount of fibrosis was quantified by picro Sirius Red ­staining. From each stained section, up to 40 digital ­photomicrographs from different areas were taken, depending upon section size. For fibrosis content, the percentage of picro Sirius Red staining in each photomicrograph was determined. For each section, staining in all photomicrographs was averaged.

Cell size was determined in transversal sections after labeling of sections with anti-dystrophin. Photomicrographs (more than five photomicrographs of different areas from each section) of anti-dystrophin labeled sections at 20× magnification were used to measure transversal cell surface area (TCSA) in square micrometer. Sodium channel expression by anti-Na_v_1.5 antibody was determined, as described previously (18).

The protein level and distribution of total Cx43 were determined in parallel sections by immunohistochemistry, as described previously (19). The protein level and distribution of non-phosphorylated Cx43 were revealed by a monoclonal mouse anti-Cx43 antibody (Zymed 13-8300). Immunostaining intensity was quantified, as described previously (11). Heterogeneity of Cx43 distribution was quantified in 1280 × 1024 pixel size photomicrographs of the Cx43 labeling. These photomicrographs were transformed into eight-bit black (Cx43) and white (background) pictures. A custom written script in Matlab (The MathWorks Inc., USA) was applied to assess the shortest distance for each Cx43 pixel to its neighboring Cx43 pixel. SD of all shortest distances of all Cx43 pixels was used as a measure of spatial Cx43 heterogeneity.

Total Cx43 protein levels were determined by Western blot analysis, as described previously (19). A Cx43-specific C-terminal antibody was utilized to visualize the unphosphorylated isoform of Cx43 (Cx43-P0) (20).

### Statistical Analysis

Data were analyzed using SPSS software (SPSS 17, Chicago, IL, USA). Statistical significance of differences was evaluated by unpaired Student’s *t*-test or ANOVA followed by *post hoc* Bonferroni test as appropriate. Arrhythmia incidence was compared with Fisher’s exact test. Two-sided *P* values <0.05 were considered statistically significant. Data are expressed as mean ± SEM.

## Results

### Echocardiography and ECG Data

After 4 weeks of pressure overload, TAC rats showed hypertrophy with preserved LV function. Diastolic LV posterior wall thickness and heart/body weight ratio increased significantly (Table S1 in Supplementary Material). Fractional shortening improved in this early compensated hypertrophic state. QRS duration was significantly longer in TAC (20.4 ± 0.38 ms) compared to sham-operated rats (17.8 ± 0.44 ms; *P* < 0.01).

### Arrhythmia Susceptibility and Conduction Velocity

**Figure 1:**
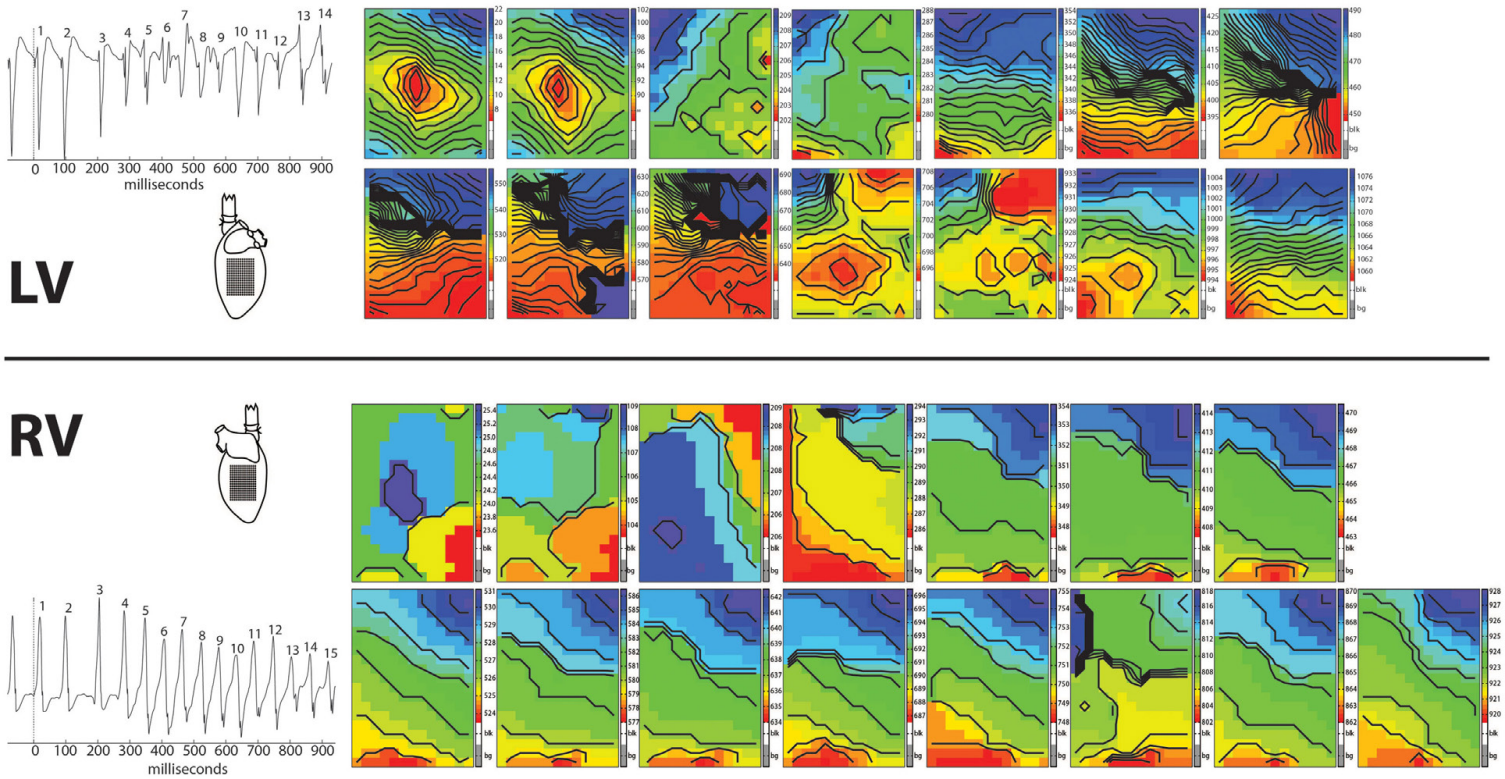
**Arrhythmia**. Epicardial activation maps of a polymorphic tachycardia induced in the left ventricle of a Langendorff perfused TAC rat heart. Simultaneously recorded activation maps of LV and RV are displayed in chronical order to show the evolution of the arrhythmia. Tachycardia was induced by one premature stimulus applied at the center of the recording electrode on LV. First two activation maps are from the last basic and the premature stimulus. Note the centrifugal spread of activation of these two maps of LV (red earliest activation, blue latest activation). Activation patterns of LV are more complex then of RV, showing multiple and varying areas of conduction block/slowing (areas of crowding isochronal lines). In contrast, activation patterns in RV show broad fronts, sweeping over the epicardium at high speed.

**Figure 2:**
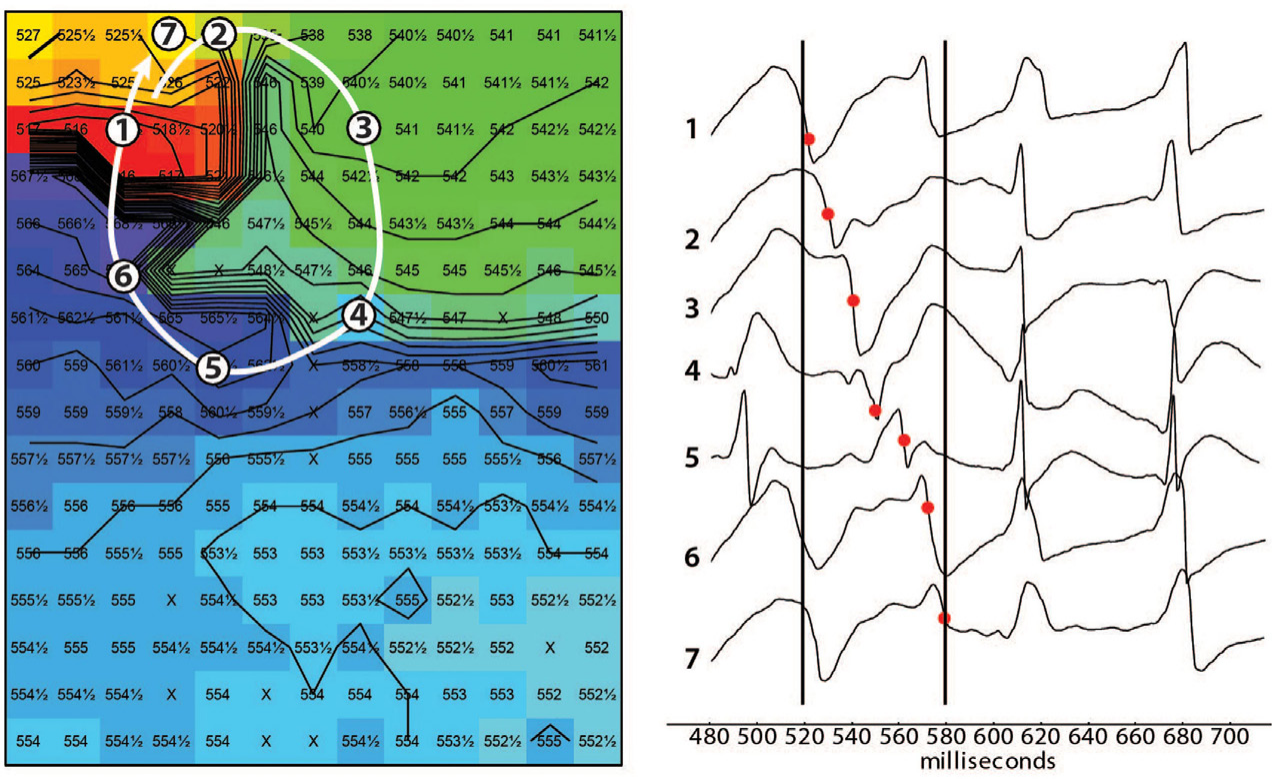
**Epicardial activation map showing reentry**. Example of reentrant activation (white arrow) at the LV epicardium occurring 520 ms after induction of a tachycardia by a single premature stimulus. Note the sequence of activation indicated by the black dots in the tracings. The following beat activates the area where reentry occurred almost simultaneously (electrograms after the right black line occur almost simultaneously). Black lines are isochronal lines at distances of 2 mm, numbers are activation times with respect to the premature stimulus that induced the tachycardia. Reentry occurred frequently but was never stable.

In 8/13 TAC rats (62%), polymorphic ventricular tachyarrhythmias (pVT) were induced by premature or burst stimulation compared to 0/13 in sham rats (*P* = 0.001). During VT, epicardial electrical activation patterns of LV during VT showed variable sites of earliest epicardial activation of consecutive beats, and altering sites of functional conduction block (Figure 1). In contrast, RV epicardial activation maps showed broad and fast activation fronts. Wandering epicardial reentrant activation was sporadically observed. Figure 2 shows a rare example of a single reentrant pathway on the LV epicardium, which starts 520 ms after induction of VT and completes at *t* = 580 ms. However, following up on this, the area where reentry occurred was activated almost simultaneously during the next beat (electrograms at electrode sites 2–7 occur almost simultaneously).

Epicardial CV longitudinal and transversal to fiber direction was not different between sham- and TAC-operated animals, neither for RV nor LV (Table 1). However, transmural CV was slowed in TAC-operated rats compared to sham-operated animals (37.6 ± 2.9 vs. 58.5 ± 3.9 cm s^−1^ respectively; *P* < 0.01). ERP in both RV and LV was longer in TAC compared to sham-operated animals, but the difference did not reach significance (Table 1).

**Table 1:** **Mapping data: data of electrical mapping study during Langendorff perfusion**.

	Sham	TAC		
		All	TAC+	TAC−
RV θ_L_ (cm s^−1^)	68.6 ± 2.4	67.1 ± 3.4	65.2 ± 4.6	70.1 ± 5.3
RV θ_T_ (cm s^−1^)	43.4 ± 2.3	39.0 ± 1.9	41.1 ± 2.5	35.6 ± 2.7
RV anisotropic ratio	1.64 ± 0.10	1.75 ± 0.09	1.60 ± 0.11	1.98 ± 0.09
RV ERP (ms)	45.4 ± 2.7	54.6 ± 3.9	60.0 ± 5.3	46.0 ± 2.4
RV DoC (cm s^−1^)	1.21 ± 0.088	1.32 ± 0.13	1.40 ± 0.20	1.20 ± 0.12
LV θ_L_ (cm s^−1^)	62.8 ± 5.7	68.9 ± 3.3	64.3 ± 4.9	74.5 ± 3.5
LV θ_T_ (cm s^−1^)	27.5 ± 2.5	27.9 ± 2.7	26.8 ± 3.2	29.2 ± 5.1
LV anisotropic ratio	2.29 ± 0.13	2.63 ± 0.20	2.48 ± 0.22	2.80 ± 0.38
LV ERP (ms)	59.1 ± 4.1	67.5 ± 3.3	65.7 ± 4.8	70.0 ± 4.5
LV DoC (cm s^−1^)	1.23 ± 0.075	1.90 ± 0.087*	**2.10 ± 0.060**^†^	1.64 ± 0.12
LV θ_TM_ (cm s^−1^)	58.5 ± 3.9	37.6 ± 2.9*	35.0 ± 4.8	40.7 ± 2.9

*RV/LV, right and left ventricle, respectively; θ_L_/θ_T_/θ_TM_, conduction velocity longitudinal, transversal, or perpendicular to epicardial fiber direction, respectively, at pacing cycle length of 150 ms; ERP, effective refractory period; DoC, dispersion of conduction*.

*P < 0.01 vs. sham; ^†^*P* < 0.01 vs. TAC−.

*Significant comparisons are highlighted in bold*.

### Factors Associated with Arrhythmogenic Remodeling

**Figure 3:**
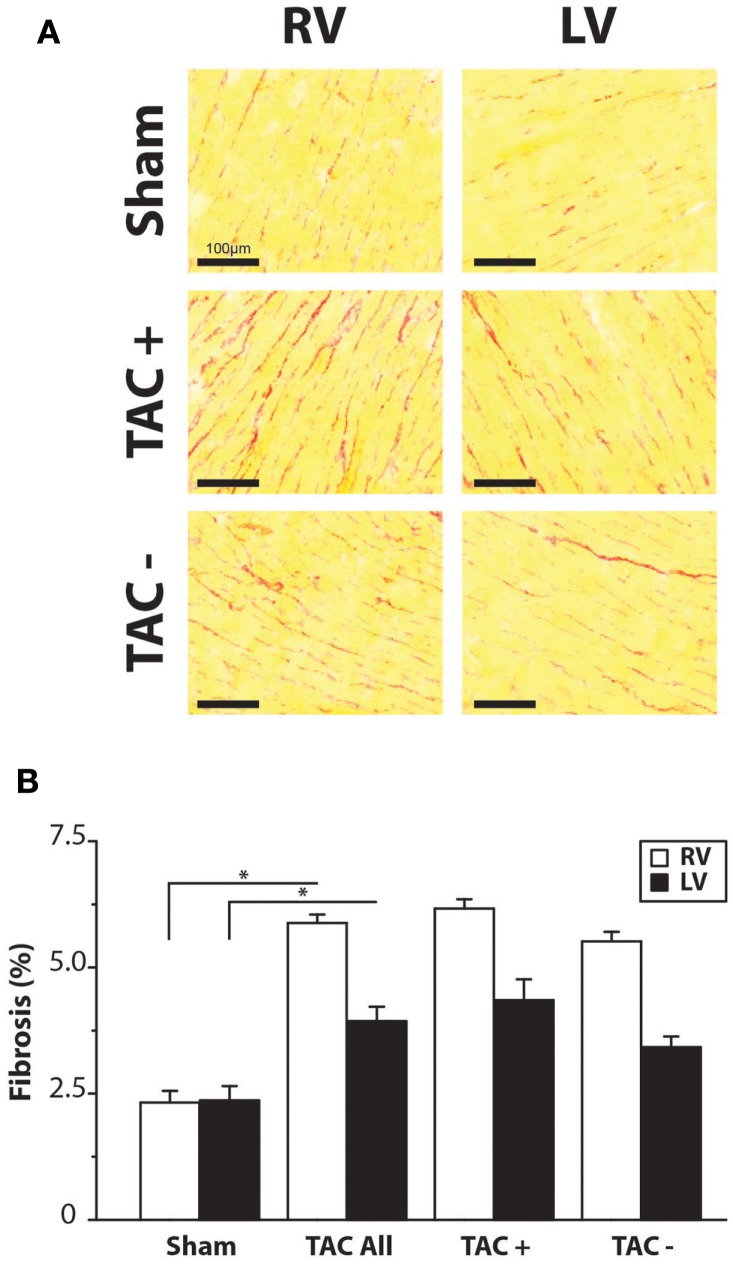
**Fibrosis**. **(A)** Representative examples of Sirius Red staining. Right and left ventricle (RV/LV) of sham and TAC rats. **(B)** Quantifications of collagen content in percentage of total slide area. Collagen content was significantly higher in TAC compared to sham animals. There was no difference between TAC+ and TAC−.

To determine parameters associated with arrhythmogeneity, we measured cell size, collagen content, sodium channel (SCN5a), and Cx43 protein levels and distribution. In TAC rats, TCSA was significantly larger in both LV (12%) and RV (11%) compared to sham rats (Table 2). The amount of collagen was significantly more abundant in LV (63%) and RV (156%) of TAC rats compared to sham-operated animals (Figure 3; Table 2). Sodium channel expression as determined by quantification of Na_v_1.5 immunofluorescence was not different between TAC and sham (Table 2).

**Table 2:** **Cellular data and fibrosis: data of cell size measured as transversal cell surface area (TCSA), percentage of interstitial fibrosis, Na_v_1.5 sodium channel, Cx43 gap junction channel, and its heterogeneity (Cx43 *het*) as derived from immunofluorescent signals**.

	Sham	TAC		
		All	TAC+	TAC−
**Cell size**				
RV (µm^2^)	473 ± 28	585 ± 36*	**516 ± 36^†^**	671 ± 32
LV (µm^2^)	473 ± 31	599 ± 40*	**515 ± 37^†^**	704 ± 26
**Fibrosis**				
RV (%)	2.3 ± 0.2	**5.9 ± 0.2^†^**	6.2 ± 0.2	5.5 ± 0.2
LV (%)	2.4 ± 0.3	**3.9 ± 0.3^†^**	4.4 ± 0.4	3.4 ± 0.2
**Nav1.5**				
RV (%)	1.3 ± 0.5	1.2 ± 0.4	0.7 ± 0.2	2.1 ± 0.9
LV (%)	1.0 ± 0.2	1.2 ± 0.2	1.0 ± 0.2	1.6 ± 0.4
**Cx43**				
RV (%)	1.9 ± 0.2	–	1.9 ± 0.2	1.8 ± 0.1
LV (%)	1.5 ± 0.2	–	1.4 ± 0.2	1.8 ± 0.2
RV het (µm)	8.1 ± 0.2	8.9 ± 0.2*	9.0 ± 0.2	8.7 ± 0.5
LV het (µm)	7.7 ± 0.2	**9.6 ± 0.4^†^**	**10.2 ± 0.4^†^**	8.6 ± 0.2

*t-Test for comparison of TAC All vs. sham; ANOVA for comparison of TAC+ vs. TAC− vs. sham*.

*Significant comparisons are highlighted in bold*.

*P < 0.05 vs. sham; ^†^*P* < 0.01 vs. TAC−.

**Figure 4:**
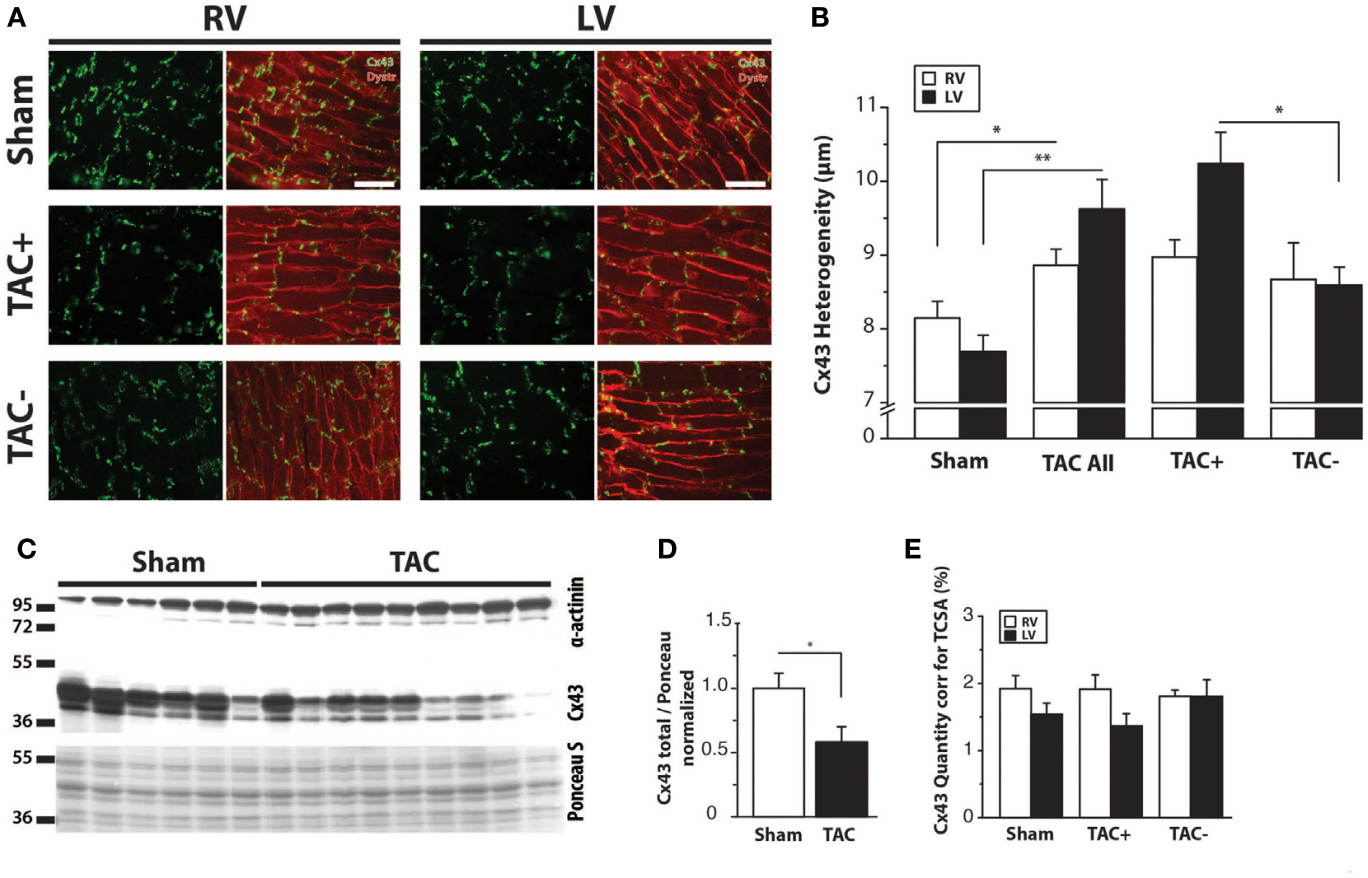
**Cx43 distribution**. **(A)** Typical examples of anti-Cx43 labeling along with merged photographs of both anti-Cx43 and anti-dystrophin labeling from right and left ventricle (RV/LV) of sham- and TAC-operated rats with or without arrhythmia susceptibility (TAC+/TAC−). Scale bar = 100 μm. **(B)** Quantifications of heterogeneity of Cx43 distribution are shown (see also Table 2). *P < 0.05 vs. same ventricle; **P < 0.01 vs. same ventricle. **(C)** Western blot showing Cx43 including the bands for different phosphorylation states in LV myocardial samples from TAC and sham rats. Ponceau S blot is for validation of equal loading. **(D)** Quantifications of total Cx43 expression, normalized for sham. **(E)** Quantifications of total Cx43 expression, normalized for cell size (TCSA, Table 2).

Immunolabeling of Cx43 protein displayed a significantly more heterogeneous spatial distribution in TAC compared to sham-operated animals (Table 2; Figures 4A,B). Some areas exhibited normal homogeneous Cx43 labeling, whereas in other regions, Cx43 labeling was heterogeneous or even nearly absent. In addition, Western blot analysis of total Cx43 protein levels revealed a lower level of Cx43 in TAC rats (Figure 4C, and quantified after normalization to total protein in Figure 4D). However, when total Cx43 protein signals in the different groups were corrected for their respective TCSA values (cell size, Table 2), this reduction could not be confirmed (Figure 4E).

**Figure 5:**
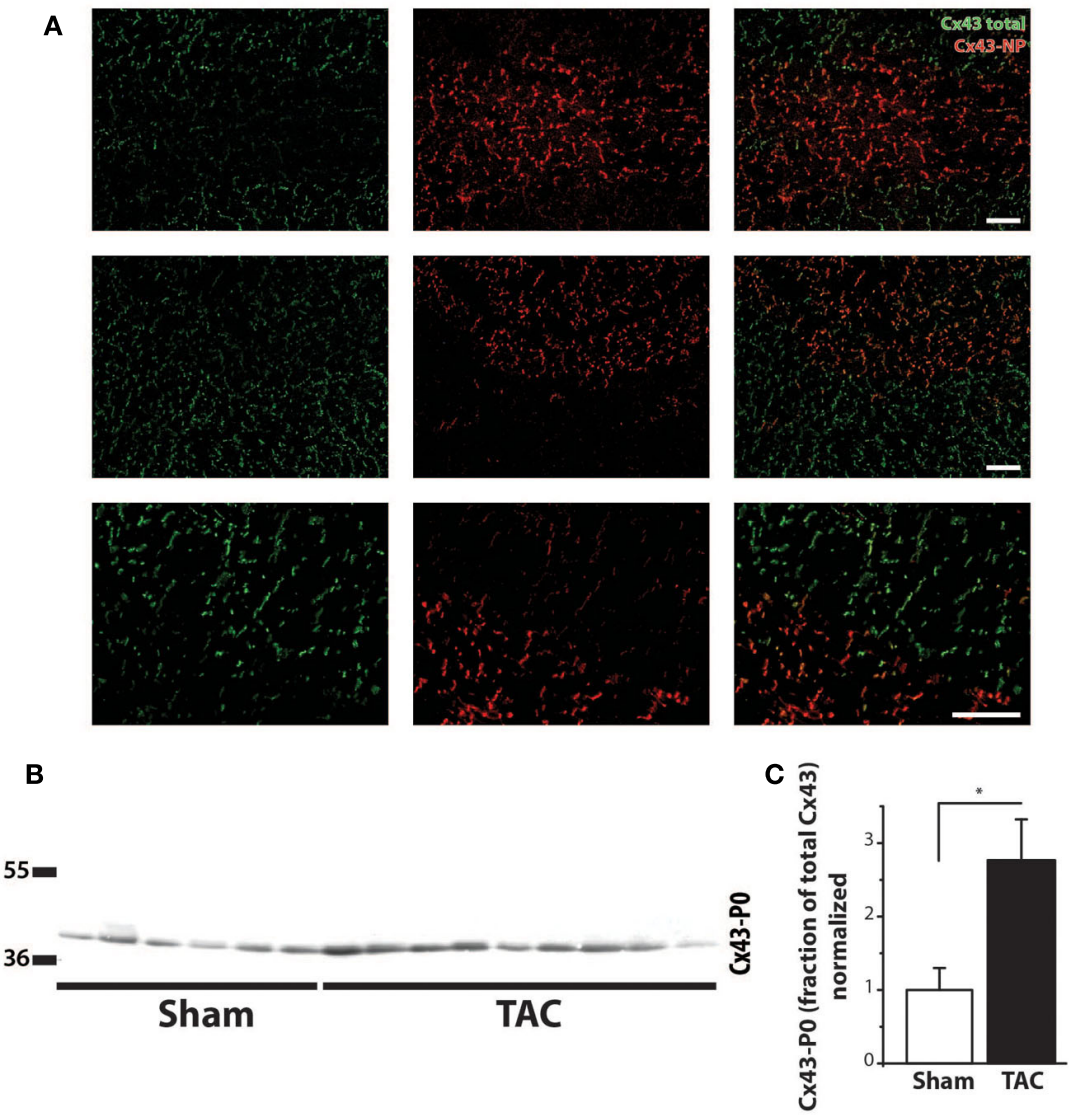
**Cx43 dephosphorylation**. **(A)** Typical examples of immunolabeling with anti-Cx43 and anti-non-phosphorylated Cx43 (Cx43-NP) along with merged photographs of both labelings from LV of two different TAC+ rats (upper two rows). Lowest row shows a higher magnification of a border zone between normal Cx43 expression and diminished Cx43 with substitution by Cx43-NP. **(B)** Western blot of non-phosphorylated Cx43 expression (Cx43-P0) in the same LV myocardial samples as in Figure 4C. **(C)** Quantifications of Cx43-NP expression, normalized to sham. Scale bar = 100 μm. *P < 0.05 vs. sham.

Regarding the heterogeneity of Cx43 remodeling, Figure 5A shows that in areas where total Cx43 signals were reduced or apparently even absent, the non-phosphorylated isoform of Cx43 (Cx43-NP) appeared still present and predominant. This observation was further substantiated *via* Western blot analysis, which revealed a significantly increased level of Cx43-NP in TAC animals (Figures 5B,C).

### Discriminative Alterations Involved in the Onset of Arrhythmogenesis

To determine the relevance of these changes for arrhythmogenesis, we compared TAC rats with (TAC+) and without (TAC−) arrhythmias. QRS duration was not different between TAC+ and TAC− rats (Table S1 in Supplementary Material). Furthermore, TAC+ rats and TAC− rats did not show any difference in ventricular function, although calculated LV mass was significantly lower in TAC+ compared to TAC− (340.7 ± 32.1 vs. 443.9 ± 27.4 μg; *P* = 0.028; Table S1 in Supplementary Material). In line with that finding, TCSA showed that cell size in LV and RV was significantly smaller in TAC+ as compared to TAC− animals (Table 2).

On the other hand, no significant difference existed in the collagen content of either LV or RV between TAC+ and TAC− animals (Table 2). The same accounted for the protein levels of total Cx43 and the sodium channel (Figure 4E; Table 2).

**Figure 6:**
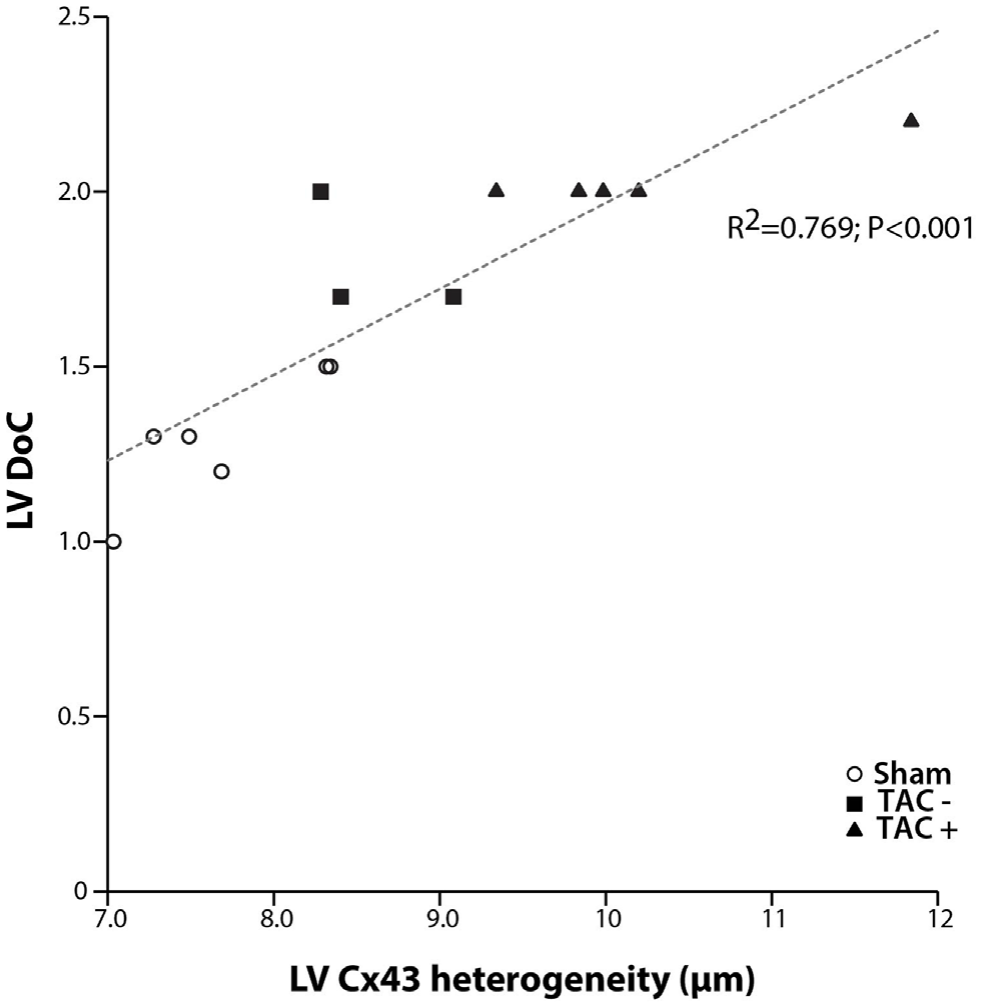
**Dispersion of conduction and Cx43 heterogeneity**. Graph showing the relation between dispersion of conduction (DoC) and Cx43 heterogeneity. A clear linear relation is shown with a high correlation coefficient (*R*^2^ = 0.769; *P* < 0.001). With increasing spatial heterogeneity of Cx43 distribution, dispersion of conduction becomes higher.

Although transmural CV was lower in TAC+ compared to TAC− animals (35.0 ± 4.8 vs. 40.7 ± 2.9 cm s^−1^, Table 1), this difference did not reach significance. On the other hand, dispersion of conduction (DoC) in LV was significantly higher in TAC hearts and did differ between TAC+ and TAC− animals, with a higher DoC in TAC+ (2.10 ± 0.060 cm s^−1^) compared to TAC− rats (1.64 ± 0.12 cm s^−1^; *P* < 0.01, Table 1). Fitting to these data on DoC, spatial Cx43 expression was most heterogeneous in TAC+, as compared to TAC− rats (*P* < 0.05; Table 2; Figure 4B). Additionally, areas with an apparently reduced Cx43 protein level showed an increased fraction of non-phosphorylated Cx43 (Figure 5A), but this shift was not significantly different between TAC+ and TAC−. Finally, in LV but not RV, dispersion in conduction was linearly correlated to the level of Cx43 heterogeneity (*R*^2^ = 0.769; *P* < 0.001; Figure 6).

## Discussion

The main and novel results from this study are 1) polymorphic tachycardias can be induced by programed stimulation from the LV in 62% of rat hearts in which compensated hypertrophy was induced by TAC. 2) Activation patterns during polymorphic tachycardias were more complex in LV than RV showing areas of conduction slowing and conduction block, unstable reentrant circuits, and multiple sites of epicardial breakthrough in LV. Wave fronts in RV were broad and propagated fast suggesting a prominent role for the LV in the detected arrhythmias. 3) Transmural conduction slowing with a generally preserved epicardial CV. 4) Heterogeneous Cx43 expression is associated with spatially dispersed impulse conduction, which enhances vulnerability to *polymorphic* ventricular tachycardias. 5) The heterogeneous Cx43 expression is correlated with a shift toward predominance of the non-phosphorylated isoform in regions with reduced Cx43 expression.

### Reentry or Focal Mechanism?

The mapping data suggest that initiation of ventricular tachycardias by premature stimulation in this rat model of compensated hypertrophy is based on a focal mechanism rather than on reentry. However, conduction delay and epicardial reentrant activation were observed in later phases of the tachycardias, which indicate that the reentry might play a role in perpetuation of the arrhythmia. No support for a reentrant mechanism in the *initiation* of the tachycardia was found, since there was no evidence for unidirectional conduction block near the site of stimulation during initiation of the tachycardia. Earliest epicardial activation of the initial ectopic beats of the VTs always occurred distant from the site of stimulation.

A focal initiation of VT may result from triggered ­activity arising from either early- or delayed-afterdepolarizations (EADs or DADs). Action potential duration was increased in the hypertrophied hearts compared to hearts of sham-operated animals, which enhances the possibility for triggered activity (21). In patients with end-stage dilated cardiomyopathy, a focal mechanism could be associated with a polymorphic ventricular tachycardia (22). In our study, the TAC hearts displayed an increased collagen deposition, a heterogeneously decreased Cx43 expression, and a reduced phosphorylation status of Cx43, which most likely results in a reduced electrical coupling. Reduced electrical coupling not only increases the possibility for reentrant arrhythmias but also favors the occurrence of focal activity (23), and, as explained, both mechanisms might have been active in this model of hypertrophy.

The occurrence of focal activity and reduced phosphorylation status of Cx43 are compatible with the observation made by Jin et al., who found afterdepolarizations as trigger for arrhythmias using optical mapping in a similar model (24).

Furthermore, we observed that following ectopic beats had longer activation times (latest − earliest activation of a beat within the recording area), which increased from 5 to 40 ms within a few beats (Figure 1). Isochrones became more crowded, indicative for a rundown of the CV. In addition, zones of conduction block arose. These changes set up the possibility for reentry, which we indeed observed in LV, as illustrated in Figure 2.

### Polymorphic VTs are Correlated to Dispersed Conduction and Heterogeneous Cx43 Expression

The high susceptibility to polymorphic VTs in rats with compensated hypertrophy was linked to increased dispersion of LV CV and heterogeneous Cx43 expression.

The relationship between the occurrence of VT and dispersion in Cx43 expression has been shown in patients (11, 12), dogs (25, 26), rabbits (6), and mice (10, 11). Heterogeneous electrical uncoupling in our model is associated with increased electrical heterogeneities in conduction and repolarization, which may create a substrate for reentry. This implies that the polymorphic character of VTs in our TAC rats is most likely due to ­inhomogeneous ­conduction, which by itself is caused by dispersed expression of Cx43. Indeed, the increased DoC in LV highly paralleled to increased Cx43 heterogeneity (*R*^2^ = 0.769, Figure 6).

It is unclear to what extent the downregulation of Cx43 or the increased levels of fibrosis potentiate the effects of heterogeneous Cx43 expression on dispersed conduction and polymorphic VTs.

Several studies in transgenic mice with reduced Cx43 have shown that a moderate and homogeneous decrease of 50% in Cx43 expression by itself does not necessarily lead to conduction impairment (27, 28). In our TAC rats, Cx43 expression was down regulated by 42%, which did not lead to significant impairment of epicardial CV. The observed increase in QRS duration is regarded to be caused by the increase in ventricular mass (Table S1 in Supplementary Material) in a similar fashion as we showed before (29). LV transmural conduction slowing was associated with increased LV fibrosis, which is in agreement to a mild reduction in transversal conduction as observed in other studies (30). In addition, the heart may become more sensitive to spatial differences in intercellular coupling in the background of other impairments in conduction parameters. It is known that the heart has “conduction reserve,” as result of which the heart is able to maintain normal conduction velocities even under conditions of moderate changes in Cx43, SCN5a, or collagen levels (18, 31, 32). Moderate changes in Cx43 or collagen, however, may reduce this conduction reserve and sensitize the heart to local differences in electrical coupling and thus to local differences in CV.

This heterogeneous conduction substrate resulted in the occurrence of pVT. Polymorphic ventricular tachycardia in the healthy, isolated rabbit heart has been shown to result from either a single or paired (“figure-of-eight”) non-stationary scroll wave (33). Polymorphic VT in patients after myocardial infarction (MI) has been shown to be triggered and possibly maintained by activity originating from the distal Purkinje system localized in the MI border zone and could successfully be abolished by radiofrequency ablation (34). In TdP, triggered activity and wandering reentrant activation have been proposed to explain its polymorphic character (35).

### Cx43 Dephosphorylation and Conduction

As mentioned before, we observed that areas with normal Cx43 expression neighbored zones with apparently almost no Cx43 immunofluorescent signal. It appeared, however, that in these areas with poor levels of total Cx43, a shift occurred toward non-phosphorylated Cx43. Phosphorylation of Cx43 is a key mechanism in its functional gating properties. In general, dephosphorylation of Cx43 leads to impaired coupling (15), which results in slowed conduction and the genesis of unidirectional block by which the susceptibility to reentrant arrhythmias is increased (16, 36). However, as described before, reduced electrical coupling may also unmask ectopic foci, which may serve as focal drivers of arrhythmias (23).

### Transversal Conduction Slowing Is Related to Increased Interstitial Fibrosis

Increased collagen amount also gives rise to reduced cell-to-cell coupling, impaired conduction, and propensity for triggered activation. Conduction slowing by (interstitial) fibrosis mainly occurs in the transversal direction, while the effect on conduction in longitudinal direction is less prominent. This is explained by the fact that interstitial fibrosis is most often oriented parallel to the fiber direction, thereby separating cardiomyocytes on the long side. Myocardial fibers are oriented parallel to the epicardium and their direction rotates over an angle of about 120° from the epicardial plane to the endocardial plane (37). Because of this fiber rotation, measurements of transversal and longitudinal epicardial conduction velocities not only reflect epicardial conduction velocities but also those of deeper layers in different fiber angles (37). However, during transmural conduction, the activation front is nearly perpendicular to fiber orientation, and most sensitive to increased levels of interstitial fibrosis. Indeed, in our model, transmural CV was significantly reduced. However, although collagen expression was significantly increased in TAC rats, no differences were observed between TAC+ and TAC− rats. As such, fibrosis was not identified as a discriminating factor explaining the observed arrhythmogeneity. As mentioned before, increased levels of fibrosis itself may contribute to a compromised conduction reserve and increased propensity for polymorphic VTs.

## Conclusion

We have shown a significant correlation between heterogeneous Cx43 expression, on one hand, and dispersed impulse conduction and enhanced vulnerability to *polymorphic* ventricular tachycardias, on the other hand. The heterogeneous Cx43 expression was furthermore associated with a shift toward the non-­phosphorylated isoform of Cx43 in the regions with reduced Cx43 expression.

## Author Contributions

Conducting the experiments: MB, ME, MS, BK, SA, HR, and TV; design of the study: MV, HR, JB, and TV; data analysis: MB, MFAB, MV, HR, JB, and TV; writing and critical revision of the manuscript: MB, MFAB, MV, HR, JB, and TV.

## Conflict of Interest Statement

The authors declare that the research was conducted in the absence of any commercial or financial relationships that could be construed as a potential conflict of interest.
