# Supplementary material for: Spatial Heterogeneity of Cx43 is an Arrhythmogenic Substrate of Polymorphic Ventricular Tachycardias during Compensated Cardiac Hypertrophy in Rats
Source: Front Cardiovasc Med. 2016 Mar 2;3:5. doi: 10.3389/fcvm.2016.00005 (PMC4773605; doi:10.3389/fcvm.2016.00005)
Supplement: Supplementary file 1 [file Table_1.PDF]

Supplemental Table 1 – *Rat characteristics:* Doppler-echocardiographic and ECG parameters of rats at 4 weeks follow-up. Data of TAC rats are subdivided into arrhythmogenic (TAC+) and non-arrhythmogenic (TAC-) rats. BW, body weight; HWBW, heart weight to body weight ratio; LVFS, LV fractional shortening; LVEDV, LV end-diastolic volume; LVAWd and LVPWd, LV diastolic anterior wall and posterior wall thickness; LVEDD, LV end-diastolic diameter; LVLd, LV diastolic length in long axis. RR interval was measured during echocardiography. *, P<0.05; **P<0.01 *vs.* sham; †, P<0.01 *vs.* TAC-. Significant comparisons are highlighted in bold.

	**Sham**	**TAC**		
		All	*TAC+*	*TAC-*
N	13	13	8	5
**Baseline characteristics**				
HWBW, g·kg^{-1}	4.93 ± 0.16	**6.50 ± 0.26****	6.78 ± 0.31	6.05 ± 0.39
LuWBW, g·kg^{-1}	4.48 ± 0.16	5.09 ± 0.27	5.10 ± 0.29	5.06 ± 0.59
Pressure gradient, mmHg		86 ± 5	90 ± 7	81 ± 9
**Echocardiography**				
CL, ms	148 ± 3	145 ± 4	143 ± 6	147 ± 4
CO, ml·min^{-1}	104 ± 9.0	126 ± 9.9	120 ± 6	134 ± 22
LVFS, %	45.7 ± 0.8	**51.8 ± 2.3***	51.3 ± 3.5	52.6 ± 2.5
LVEDV, µl	189.2 ± 3.9	183.8 ± 6.9	184.0 ± 9.2	183.4 ± 11.6
LVESV, µl	45.4 ± 1.8	35.8 ± 5.7	37.6 ± 8.7	32.8 ± 6.3
LVAWd, mm	0.94 ± 0.02	**1.13 ± 0.06***	1.06 ± 0.09	1.25 ± 0.05
LVAWs, mm	1.04 ± 0.04	1.22 ± 0.08	1.10 ± 0.10	1.40 ± 0.08
LVPWd, mm	1.16 ± 0.05	1.24 ± 0.07	1.14 ± 0.09	1.42 ± 0.09
LVPWs, mm	1.18 ± 0.05	1.33 ± 0.08	1.29 ± 0.13	1.37 ± 0.08
LV mass (AL)	308.9 ± 11.6	**380.4 ± 26.0***	**340.7 ± 32.1†**	443.9 ± 27.4
**ECG, all ms**				
RR	155.2 ± 9.5	159.6 ± 5.9	162.2 ± 8.6	154.9 ± 7.4
QRS	17.80 ± 0.44	**20.42 ± 0.38****	20.10 ± 0.41	20.97 ± 0.74
